# Gut microbiome dynamics of patients on dialysis: implications for complications and treatment

**DOI:** 10.3389/fphar.2025.1470232

**Published:** 2025-04-25

**Authors:** Changlin Li, Xiaomeng Lin, Yuting Li, Jiamin Duan, Xudong Cai

**Affiliations:** ^1^ Department of Nephrology, Ningbo Municipal Hospital of Traditional Chinese Medicine (TCM), Affiliated Hospital of Zhejiang Chinese Medical University, Ningbo, China; ^2^ Ningbo Institute of Chinese Medicine Research, Ningbo Municipal Hospital of Traditional Chinese Medicine (TCM), Affiliated Hospital of Zhejiang Chinese Medical University, Ningbo, China

**Keywords:** hemodialysis, peritoneal dialysis, gastrointestinal microbiome, uremic toxins, probiotics, prebiotics, synbiotics, biological products reduces endogenous antioxidant levels

## Abstract

The gut microbiome plays a significant role in dialysis. As disease progresses, the choice of dialysis method and dietary habits change, and the diversity and richness of the gut microbiome in patients on dialysis change as well. The uremic toxins produced exacerbate inflammatory responses and oxidative stress, leading to markedly different incidence rates of complications such as cardiovascular disease and dialysis-associated peritonitis among patients on dialysis. The intake of probiotics, prebiotics, synbiotics, and natural medicines during daily life can regulate the gut microbiome, reduce the production of uremic toxins in patients on dialysis. This review found that the occurrence of complications in dialysis patients is related to changes in the gut microbiome and the accumulation of uremic toxins. The use of probiotics, prebiotics, synbiotics, and natural medicines can improve these conditions and reduce the incidence of dialysis-related complications.

## 1 Introduction

Dialysis treatment, a common therapeutic method for patients with renal failure, removes excess fluid and uremic toxins (Himmelfarb et al., 2020). In 2022, the Chinese National Renal Data System included 985,000 patients on dialysis in China, with 844,000 on hemodialysis (85.7%) and 141,000 on peritoneal dialysis (14.3%).

The human gut harbors a vast number of functionally diverse gut microbes, and the composition and functional dysregulation of these microbes are closely linked to kidney disease (Lynch and Pedersen, 2016). In patients on dialysis, accumulated uremic toxins like indoxyl sulfate (IS) and p-cresol sulfate (PCS) disrupt the intestinal microenvironment, promoting pathogenic bacterial growth while inhibiting beneficial bacteria, including those producing short-chain fatty acids (SCFAs). Because dialysis methods vary, the gut microbiome of patients on peritoneal dialysis differs from that of patients on hemodialysis. Patients who undergo peritoneal dialysis experience an increased relative abundance of *Proteobacteria*, whereas patients who undergo hemodialysis experience recovery of beneficial bacteria in the gut (Simões-Silva et al., 2018). Inadequate dialysis can lead to retention of uremic toxins, which may induce complications such as cardiovascular disease (CVD) and peritonitis associated with peritoneal dialysis, thus negatively affecting the quality of life of patients (Wang X. et al., 2020; Bello et al., 2022b).

A correlation exists between chronic inflammation caused by gut microbiome dysbiosis and renal function deterioration of patients with advanced kidney disease (Ebert et al., 2021). Beneficial metabolites such as SCFAs have anti-inflammatory effects; for example, they prevent uremic toxins from entering the bloodstream through the gut and protect the cardiovascular system (Xiong et al., 2022; Pryde et al., 2002). Protein-bound uremic toxins, such as IS, PCS, and trimethylamine N-oxide (TMAO), accumulate in the blood of patients on dialysis owing to the increased permeability of the gut mucosa and incomplete toxin removal, inducing other complications (Pryde et al., 2002; Vaziri et al., 2016; Vanholder et al., 2021; Xiong et al., 2022; Dehghan Niestanak and Unsworth, 2023). For instance, in patients on hemodialysis, increased risk of blood infections is often associated with the *Escherichia* coli-Shigella complex and *Klebsiella*, while cardiovascular complications are linked to the accumulation of *Clostridium*, Parvimonas, TMAO, IS, and PCS. Additionally, renal anemia is frequently correlated with decreased *Neisseria*. TMAO promotes peritoneal dialysis fluid-induced inflammatory cell infiltration and inflammatory cytokine production in patients on peritoneal dialysis, while an increase in ubacterium eligens in the gut and Devosia in the blood is often associated with cardiovascular disease in PD patients. Similarly, IS lead to oxidative stress, upregulation of genes related to IL-1β and COX2A, and promotion of inflammation in patients on dialysis; TMAO has a pro-atherosclerotic effect that leads to cardiovascular complications and an accelerated occurrence of peritonitis associated with peritoneal dialysis (Tang et al., 2022).

Currently, the incidence rates of cardiovascular disease and peritonitis related to dialysis remain high, and traditional treatment methods have exhibited limited effectiveness. This article summarizes recently discovered therapeutic methods such as the use of probiotics and cellulose that actively regulate the gut microbiome through microbial mixtures (David et al., 2014; Shoaie et al., 2015; Simões-Silva et al., 2018) by reducing the production of uremic toxins and alleviating systemic inflammatory responses and oxidative stress, thereby delaying the occurrence of hemodialysis-related blood infections, peritoneal dialysis-associated peritonitis, and cardiovascular complications in patients on dialysis (Table 1).

**TABLE 1:** Intestinal flora and uremic toxin changes and dialysis complications.

Dialysis method	Complication	Intestinal flora or uremic toxin change	Pathological process and results	Treatment	References
Hemodialysis	Blood infection	*Escherichia* coli-Shigella complex and *Klebsiella* increased	Increased risk of bacteremia and increased antibiotic resistance	Curcumin reduces PCS plasma levels, alleviates inflammation, and lowers infection risk	Shi et al. (2022); Wu et al. (2023a); Salarolli et al. (2021)
	Hyperphosphatemia	*Clostridium* and Parvimonas were enriched	Accelerated production and entry of phosphate into the blood, aggravated inflammatory response		Zhang et al. (2022)
	Cardiovascular disease	Increased TMAO formation	Induced vascular inflammation, caused vascular calcification, and increased risk of cardiovascular death		He et al. (2022a); Zhang et al. (2020)
		Increased IS and PCS formation	Associated with endothelial dysfunction, increased atherosclerosis risk, and endothelial cell toxicity	Intake of resistant starch or consumption of extruded sorghum breakfast meal and unfermented probiotic dairy beverage can reduce levels of uremic toxins like IS and PCS	Kemp et al. (2021); Khosroshahi et al. (2019); Esgalhado et al. (2018); Esgalhado et al. (2020); Lopes et al. (2019)
	Renal anemia	Reduced *Neisseria*	Regulated nutritional status and parathyroid function	Galactomannan, resistant dextrin, fructooligosaccharides, or starch dietary fiber supplements improve iron metabolism, regulate bone marrow hematopoiesis, and alleviate anemia in kidney disease patients	Zhu et al. (2022); Li et al. (2022); Freitas et al. (2012); Huang et al. (2018);
Peritoneal dialysis	Peritoneal dialysis-related peritonitis	Increased TMAO formation	Promoted peritoneal dialysis fluid-induced inflammatory cell infiltration and inflammatory cytokine production and directly induced peritoneal mesothelial cell necrosis		He et al. (2022b)
	Cardiovascular disease	Eubacterium eligens group in the gut and Devosia in the blood increased	Affected severity of vascular calcification	*Lactobacillus* casei Zhang increases beneficial SCFA-producing bacteria, activates proliferator-activated receptor-γ, suppresses nuclear factor-κB pathways, reduces macrophage infiltration, inhibits M1 polarization, and decreases inflammatory cytokines in peritoneal dialysis effluent, alleviating peritoneal inflammation and preventing fibrosis progression FuShen granules delay peritoneal fibrosis progression by inhibiting IL-6, transforming growth factor-β1, and connective tissue growth factor expression	Luo et al. (2021); Merino-Ribas et al. (2022); Wu et al. (2023b); Lin et al. (2021b)

TMAO, trimethylamine N-oxide; IS, indoxyl sulfate; PCS, p-cresol sulfate; SCFAs, short-chain fatty acids.

## 2 Characteristics of the gut microbiome in patients on hemodialysis

The survival time of patients on hemodialysis is closely related to the diversity and richness of the gut microbiome. The gut microbiome of deceased patients often exhibits a significant decrease in the relative abundance of SCFA-producing bacteria, such as *Succinivibrio* and *Anaerostipes* (Lin T.-Y. et al., 2021). The increased abundance of *Actinobacteria* as well as the increase abundance of *Bacteroidia* in patients on hemodialysis may indicate disease progression because *Bacteroidia* includes genes for tryptophan indole lyase and tyrosine phenol-lyase, which are closely related to the synthesis of TMAO and IS, which are uremic toxins associated with a poor prognosis (Luo et al., 2021; Li et al., 2023).

### 2.1 Factors that affect the gut microbiome in patients on hemodialysis

**FIGURE 1:**
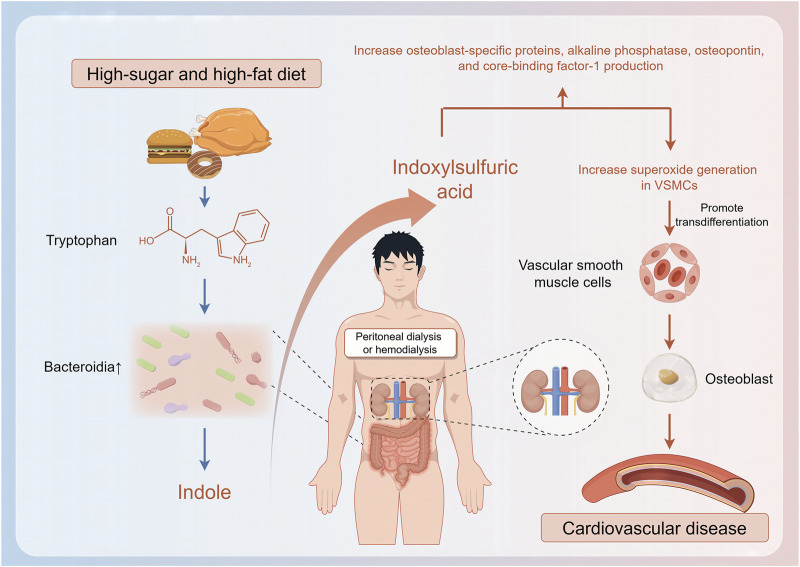
Relationship between gut microbiome changes and hemodialysis complications.

Although changes in the characteristics of the gut microbiome of patients on hemodialysis are related to disease progression, they are also related to nutritional status, which is associated with body composition and dietary habits (Shivani et al., 2022). Patients with normal weight obesity (those with a normal body mass index and high body fat percentage rather than a normal body mass index and normal body fat percentage) have a significantly lower relative abundance of butyrate-producing bacteria such as *F. prausnitzii* and *Coprococcus*, experience frequent inflammatory responses, and are more likely to subsequently develop malnutrition; therefore, these patients’ prognosis is often poor (Molnar et al., 2010; Lin et al., 2020). Similarly, protein-energy wasting also affects the levels of butyrate-producing bacteria such as *Faecalibacterium prausnitzii* in patients on hemodialysis, resulting in significantly higher levels of inflammatory factors than those in patients with a normal nutritional status (Lin and Hung, 2021). The abundance of *Haemophilus* and that of *Haemophilus parainfluenzae* are relatively lower in patients on hemodialysis with a higher plant-based diet index, which reflects high consumption of fiber, vitamins, and unsaturated fatty acids; this type of diet can reduce the production of IS by inhibiting the expression of microbial enzyme TnaA. Patients with a lower plant-based diet index, which reflects the consumption of substantial unhealthy foods such as animal fats and sweets, experience proliferation of bacteria related to IS and p-cresol, which can trigger formation of reactive oxygen species, induce transformation of vascular smooth muscle cells to osteoblast phenotypes, and increase CVD risk (Lu et al., 2021; Stanford et al., 2021). Dietary habits, dialysis cycles, health status, and residual renal function-related factors affect the structure of the gut microbiome, thus leading to accumulation of uremic toxins, which, in turn, affect the gut microbiome. For instance, accumulation of IS and PCS may worsen dysbiosis of the gut microbiome and induce complications (Eloot et al., 2017; Li et al., 2019; Zhang P. et al., 2023) (Figure 1).

**FIGURE 2:**
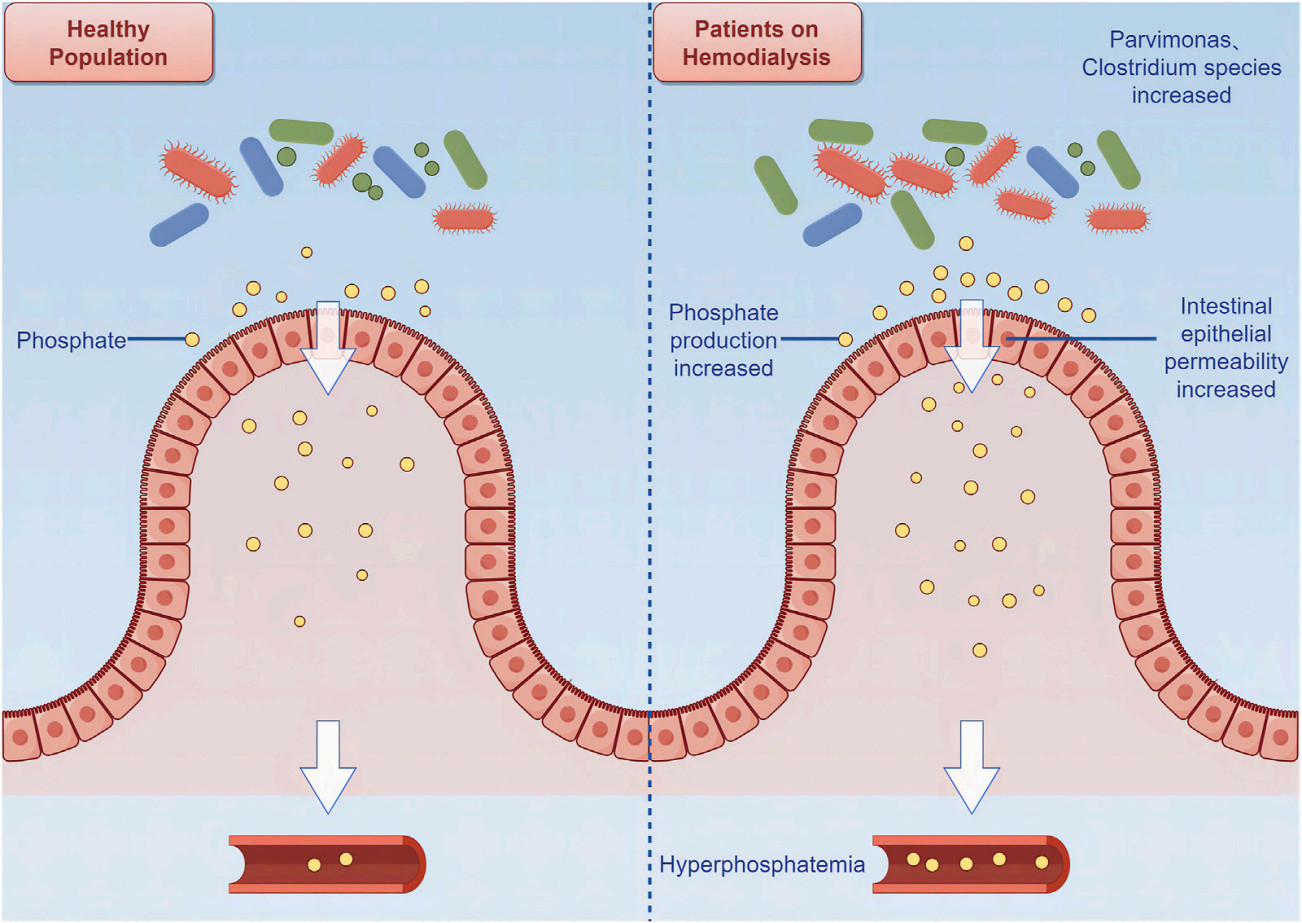
Relationship between intestinal flora and cardiovascular disease among patients on hemodialysis.

Many patients on hemodialysis experience inadequate control of blood phosphate levels, which increases their risk of cardiovascular death (Miao et al., 2018). Accumulation of phosphate in patients on hemodialysis is related to increased production, abnormal transformation, absorption, and utilization of phosphate in the gut (Larsson et al., 2018; Zhou et al., 2021). *Clostridium* species in the gut of patients on hemodialysis gradually accumulate with disease progression, thus accelerating phosphate production in the gut. Additionally, accumulation of *Parvimonas* may exacerbate inflammatory responses and increase intestinal epithelial permeability, thus making it easier for phosphate to enter the bloodstream and ultimately leading to an increased incidence of CVD (Lau et al., 2015; Miao et al., 2018) (Figure 2).

Blood infections are a major cause of hospitalization, morbidity, and death among patients on hemodialysis; therefore, controlling bloodstream infections has always been a focus of the Centers for Disease Control and Prevention (Gupta et al., 2013). Facilities The increased abundance of *Escherichia coli*–*Shigella* complex and that of *Klebsiella* species in the gut of patients on hemodialysis increase the risk of bacteremia, and increased antibiotic resistance further increases the likelihood of infection among patients on hemodialysis (Shi et al., 2022). Dysbiosis of the gut microbiome is considered a notable factor associated with the cardiovascular risk of patients with end-stage renal disease (ESRD) and includes the promotion of vascular calcification by uremic toxins, which can increase the incidence of CVD (Wang et al., 2011; Tang et al., 2015). TMAO, which originates from dietary carnitine, choline, and betaine through the action of the gut microbiome, accumulates in patients on hemodialysis. Accumulation of TMAO is associated with age, long dialysis durations, increased levels of plasma intact parathyroid hormone, and comorbid diabetes, which are independent risk factors for aortic calcification. Additionally, TMAO can promote vascular inflammation by activating the NLRP3 inflammasome and nuclear factor-κB pathway, thus inducing IL-1β upregulation in vascular smooth muscle cells, causing vascular calcification, and increasing the risk of cardiovascular death (Cho and Caudill, 2017; Zhang et al., 2020; Thomas and Fernandez, 2021; He L. et al., 2022).

Patients on hemodialysis often have renal anemia, which is characterized by a relative or absolute deficiency of erythropoietin (EPO) or the accumulation of uremic toxins and interferes with red blood cell production and metabolism (Li et al., 2022). The gut microbiome has an important regulatory effect on the hematopoietic function of patients on hemodialysis. Patients on hemodialysis who are hyporesponsive to EPO have poor interconnections between gut microbiome communities. *Neisseria* species can regulate the nutritional status and parathyroid function, thus affecting the responsiveness to EPO in patients on hemodialysis, enabling the prediction of a hyporesponse to EPO, and potentially serving as a possible treatment target for renal anemia (Zhu et al., 2022).

## 3 Characteristics of the gut microbiome in patients on peritoneal dialysis

Globally, 11% of patients on dialysis undergo peritoneal dialysis, which is a convenient and safe treatment; however, these patients exhibit reduced diversity in their gut microbiome than those in healthy individuals (Bello et al., 2022a; Teitelbaum and Finkelstein, 2023). Overall, the relative abundance of *Firmicutes* and that of *Actinobacteria* in patients on peritoneal dialysis are lower than those in healthy individuals; however, the abundance of Enterobacteriaceae is increased (Szeto et al., 2006; Crespo-Salgado et al., 2016; Wu et al., 2020). The increased abundance of Enterobacteriaceae is associated with a higher incidence of peritoneal dialysis-related peritonitis. Additionally, the enrichment of bacteria that produce p-cresol and IS can lead to gut barrier dysfunction and systemic inflammation (Bao et al., 2022).

### 3.1 Factors that affect the gut microbiome in patients on peritoneal dialysis

Distribution of the gut microbiome in patients on peritoneal dialysis is influenced by many factors other than disease progression, such as cohabitation with family members, diet, duration of peritoneal dialysis, exposure to peritoneal glucose, estimated glomerular filtration rate, and physical activity (Shivani et al., 2022). Long-term cohabitation with family members can lead to similar gut microbiome structures. The abundance of *Proteobacteria*, a potential marker of gut microbiome dysbiosis, does not vary between patients on peritoneal dialysis and healthy family members; this indicates that long-term hospitalization may exacerbate changes in the gut microbiome of patients on peritoneal dialysis (Teixeira et al., 2023). The gut microbiome structures of patients on peritoneal dialysis with longer dialysis durations, more exposure to peritoneal glucose, and poorer residual renal function are different from those of other patients; furthermore, such patients have reduced SCFA production, as evidenced by the significantly lower concentrations of isobutyric acid and isovaleric acid in their feces (Jiang et al., 2021).

### 3.2 Relationship between gut microbiome changes and peritoneal dialysis complications

Peritonitis associated with peritoneal dialysis, which is one of the most common complications experienced by patients on peritoneal dialysis, often leads to the discontinuation of peritoneal dialysis, transition to hemodialysis, extended hospital stays, and even death (Chaudhary, 2011; Perl et al., 2020; Boudville et al., 2012). The inflammatory response and macrophage involvement in the peritoneum are important pathological mechanisms of peritoneal fibrosis. During peritoneal dialysis, the peritoneum is exposed to non-self-peritoneal dialysis fluid, thus leading to overproduction of inflammatory bodies, such as caspase-1, IL-1β, and IL-18, and increased expression of macrophage CD44 levels. This exacerbates peritoneal tissue fibrosis and limits the effectiveness of peritoneal dialysis (Kadoya et al., 2023). Dysbiosis of the gut microbiome in patients on peritoneal dialysis can damage gut barrier function, increase the host’s susceptibility to pathogen invasion, and increase the risk of peritonitis (Wang et al., 2012; Zhou et al., 2022). TMAO is closely related to systemic inflammation and peritonitis associated with peritoneal dialysis. Animal experiments have shown that TMAO can significantly promote inflammation, cell infiltration, and the production of inflammatory cytokines, such as IL-6, induced by peritoneal dialysis fluid. *In vitro* experiments have shown that TMAO can directly induce necrosis of peritoneal mesothelial cells and significantly increase the production of P-selectin induced by the expressions of tumor necrosis factor-α and CCL2, which are induced by high glucose in endothelial cells (Helmke et al., 2019; Zhang et al., 2022). Patients on peritoneal dialysis with higher TMAO levels have increased C-reactive protein and b2-M levels, thus increasing the risk of systemic inflammation and leading to higher mortality rates (Wang et al., 2003).

Vascular calcification is a recognized risk factor for CVD in patients on peritoneal dialysis. A multicenter, prospective cohort study performed in China showed that the higher the blood calcium compliance rate, the lower the risk of coronary artery calcification progression (Zhang H. et al., 2023). The *Eubacterium eligens* group in the gut and *Devosia* in the blood may be related to the severity of vascular calcification because they affect circulating sCD14, suggesting that the gut microbiome may have a monitoring effect on the occurrence of CVD in patients on peritoneal dialysis (Merino-Ribas et al., 2022).

### 3.3 Gut microbiome-related treatment for patients on hemodialysis

Clearing protein-bound uremic toxins that are mainly excreted through renal tubular secretion is difficult with traditional hemodialysis (van Gelder et al., 2020). According to the United States Renal Data System, the main cause of death of patients on hemodialysis is CVD, such as arrhythmia or sudden cardiac death. CVD accounts for approximately 52.2% of deaths of patients on hemodialysis. Among protein-bound uremic toxins (PBUT), indoxyl sulfate (IS) and p-cresyl sulfate (pCS) are closely associated with endothelial dysfunction, an elevated risk of atherosclerosis, and endothelial cell toxicity. Probiotics, prebiotics, synbiotics, and natural products may play a significant role in modulating the concentration of uremic toxins in the body. (Lekawanvijit et al., 2012).

## 4 Probiotics, prebiotics, and synbiotics for uremic toxins in patients on hemodialysis

The therapeutic effects of probiotics, prebiotics, and synbiotics on patients on hemodialysis include the reduction of uremic toxins, such as PCS and endotoxins, thereby delaying inflammation, reducing inflammatory indicators such as C-reactive protein and IL-6, and improving the antioxidant capacity (Koppe et al., 2015; Andrade-Oliveira et al., 2019; Rysz et al., 2021). The different types (strain specificity), different intake times, and different doses of probiotics, prebiotics, and synbiotics result in significantly different uremic toxin clearance and complication prevention rates (McLoughlin et al., 2017; Cunningham et al., 2021; Cooper et al., 2023). Probiotics, prebiotics, and synbiotics have different pathways that allow the regulation of uremic toxins; among them, prebiotics are relatively significant to the improvement of uremic toxins (Nguyen et al., 2021).

The World Health Organization and the United Nations Food and Agriculture Organization define probiotics as “live microorganisms that, when consumed in adequate amounts, can confer health benefits to the host.” (Joint FAO WHO Working Group on Drafting Guidelines for the Evaluation of Probiotics in Food ed, 2006) The improvement of uremic toxins in patients on hemodialysis is mainly attributable to the adjustment of the gut microbiome composition; for example, increasing the abundance of SCFA-producing bacteria and reducing the abundance of uremic toxin-producing bacteria can improve the levels of uremic toxins in patients on hemodialysis (Zhang Y. et al., 2023). Optimization of the gut microbiome with administration of probiotics to patients on hemodialysis may require long-term supplementation, because short-term supplementation may not be effective (Borges et al., 2019). A comparison of 3 months of treatment comprising *Streptococcus thermophilus*, *Lactobacillus acidophilus*, and *Bifidobacteria longum* and 3 months of placebo treatment showed no improvement in the gut microbiome of patients on hemodialysis; therefore, reducing the uremic toxin levels and inflammatory indicators of these patients is challenging (Borges et al., 2018). After 6 months of treatment comprising Bifico (a mixture of viable bacteria including *Enterococcus faecalis*, *Bifidobacterium longum*, and *L. acidophilus*), the abundance of the Bacteroidaceae family and that of the Enterococcaceae family increased, and the abundance of dominant *Firmicutes* decreased, thus improving the concentrations of indole-3-acetic acid and acetic acid-O-glucuronide (Liu et al., 2020). Additionally, the inability of probiotics to improve the gut microbiome in patients on hemodialysis may be attributable to changes in the gut biochemical environment, such as the massive proliferation of urease-producing bacteria that leads to the production of large amounts of urea nitrogen, thus creating a vicious cycle of gut environment deterioration (Vaziri et al., 2016). The reproduction of beneficial bacteria requires a suitable gut environment; however, patients on hemodialysis have a poor gut environment. These findings provide insights that can aid in the development of probiotic-related drugs.

Prebiotics are indigestible food components that selectively stimulate the growth and activity of probiotics; therefore, they have a beneficial effect on the host (Bindels et al., 2015). In healthy individuals, probiotics have a strong ability to regulate the gut microbiome; however, adjustments of the gut microbiome structure in patients on hemodialysis with the use of prebiotics are not obvious. Patients on hemodialysis must be cautious of increased phosphorus and potassium levels; consequently, their intake of dietary fiber, mainly from grains and vegetables, is less than that of the general population. However, insufficient intake of dietary fiber reduces the usable carbohydrate substrates for gut microbes. Therefore, alternative prebiotics are required. Resistant starch is the part of starch that cannot be digested and absorbed by the small intestine (Sajilata et al., 2006). Intake of resistant starch can reduce the levels of some uremic toxins, such as serum creatinine, IS, and PCS. Further, it reduces the values of inflammatory factors, such as IL-6, alleviates inflammatory responses, and improves gut barrier function (Esgalhado et al., 2018; Khosroshahi et al., 2019; Kemp et al., 2021). Resistant starch can also relieve oxidative stress in patients on hemodialysis by increasing Nrf2 mRNA and NQO1 protein expressions and reducing plasma IS, resulting in fewer adverse outcomes (Esgalhado et al., 2020). Although cellulose can significantly improve the gut microbiome and alleviate uremic toxins, it may not fully exert its beneficial effects and could increase production of uremic toxins if it cannot be effectively fermented in the human gut; therefore, the choice of prebiotics is very important. For example, a randomized controlled trial found that bean hull fiber supplementation increased dietary fiber intake by 9 g/d but did not reduce serum levels of PCS, IS, and TMAO (which are uremic toxins) in patients on hemodialysis. In contrast, wheat bran supplementation reduced serum levels of PCS, IS, and TMAO in patients on hemodialysis (de Vries et al., 2015; Fatani et al., 2023). Similarly, sufficient inulin supplementation is necessary to improve the gut microbiome and uremic toxin levels of patients on hemodialysis (Biruete et al., 2021). Renal anemia in patients on hemodialysis may be caused by reduced EPO production or the accumulation of uremic toxins, leading to a shortened lifespan of red blood cells. The use of galactomannan, resistant dextrin, fructooligosaccharides, or starch dietary fiber supplements can improve iron metabolism and increase the abundance of SCFA-producing bacteria, such as *Lactobacillus* and Lactobacillaceae, in the gut (Freitas et al., 2012; Huang et al., 2018). This can lead to increased serum butyrate levels and regulate the bone marrow hematopoietic capacity, thus improving hemoglobin levels and alleviating anemia in patients with kidney disease (Li et al., 2022). Although probiotics have the potential to be toxic, dietary fiber supplementation has no significant side effects and is a suitable adjunct treatment that can reduce uremic toxins and alleviate the complications of chronic kidney disease in patients on hemodialysis.

Synbiotics comprise a combination of specific probiotics and prebiotics and are beneficial because they allow colonization of probiotics in the gut. Therefore, synbiotics have the advantages of both probiotics and prebiotics. For patients on hemodialysis, synbiotics can reduce uremic toxins such as IS and PCS in the blood and control inflammatory factors and oxidative stress biomarkers (Soleimani et al., 2017; Rocchetti et al., 2020). Supplementation with extruded sorghum breakfast meal and an unfermented probiotic dairy beverage can reduce PCS and IS in the serum of patients on hemodialysis, promote the production of SCFAs in the gut, reduce urea, reduce damage of the gut mucosa, and delay the progression of kidney disease (Lopes et al., 2019). The use of synbiotic gel for 2 months has been shown to promote the colonization of *Bifidobacterium* (Cruz-Mora et al., 2014). Some synbiotic supplements may increase the levels of indole and parathyroid hormone in patients on hemodialysis, possibly owing to increased indole-3-acetic acid levels, which could induce the production of parathyroid hormone (Mirzaeian et al., 2020). Although the therapeutic effects of probiotics, prebiotics, and synbiotics on the gut microbiome structure and increased levels of uremic toxins of patients on hemodialysis have been verified for some populations, large-scale clinical application is not possible until long-term studies including multiple populations, multiple centers, and randomized controlled trials can provide comprehensive supporting evidence.

## 5 Natural medicines for targeted improvement of the gut microbiome and uremic toxins

Natural medicines include metabolic products of plants, animals, and microorganisms that play important roles in protecting and maintaining human health (Li et al., 2021). Compared with the broad effects of probiotics and prebiotics that result in the improvement and clearance of the gut microbiome and uremic toxins, many natural medicines have relatively specific clearance effects on certain gut bacteria and uremic toxins, thereby improving the quality of life of patients on hemodialysis and reducing the incidence of complications. Curcumin can strengthen the gut barrier function of patients on hemodialysis, reduce PCS plasma levels, alleviate inflammatory responses, lower the risk of infection, and delay disease progression (Salarolli et al., 2021). Many natural medicines work by inhibiting the synthesis enzymes of certain uremic toxins or improving the gut microbiome, thus affecting the production of uremic toxins. Isoquercitrin can regulate the electron transport chain of gut bacteria, inhibit the activity of complex I, and weaken the establishment of H proton potential, thus affecting tryptophan transport, reducing the biosynthesis of indole, and subsequently reducing the production of IS. Additionally, isoquercitrin can reduce the abundance of *E. coli*, which can produce indole to some extent (Wang Y. et al., 2020).

Patients with ESRD who are on dialysis commonly struggle with constipation. Although nuts are a high-fiber food, they have not been considered a treatment for constipation in such patients because of their high potassium and phosphate contents. However, recent research by Lambert et al. revealed that daily almond consumption for 4 weeks did not increase blood potassium or phosphorus levels and, more importantly, resulted in an improvement in constipation symptoms, suggesting that nuts could be a beneficial treatment option for constipation in patients on dialysis (Lambert et al., 2020).

## 6 Peritoneal dialysis-related gut microbiome treatment

According to the United States Renal Data System, similar to hemodialysis, the main cause of death associated with peritoneal dialysis is CVD, accounting for 48.6% of all deaths among these patients. However, because of the specificity of peritoneal dialysis procedures, residual dialysate is a unique cause of cardiovascular death among these patients (Krediet and Balafa, 2010). Peritoneal dialysis-related peritonitis is closely related to the high mortality risk in these patients and often leads to a permanent transition to hemodialysis (Perl et al., 2020). Probiotics, prebiotics, and synbiotics can reduce the levels of CVD-related uremic toxins such as endotoxin and p-cresol, lower the mortality rate associated with CVD, and improve the gastrointestinal symptoms and quality of life of patients on peritoneal dialysis (March et al., 2020).

### 6.1 Preventive effects of probiotics, prebiotics, and synbiotics on cardiovascular events and peritonitis

The colonization of probiotics is more easily achieved in patients on peritoneal dialysis than it is in patients on hemodialysis because peritoneal dialysis has a weaker clearance effect on probiotics.

**FIGURE 3:**
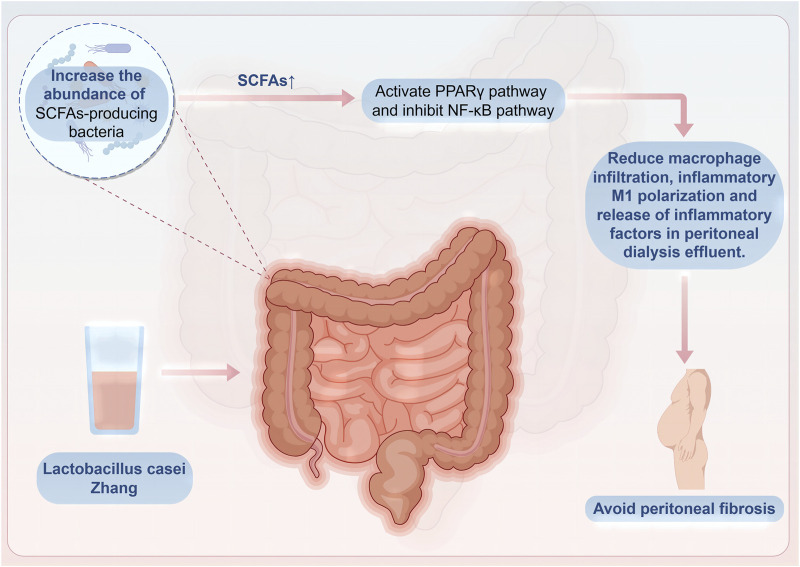
*Lactobacillus* casei Zhang prevents peritoneal fibrosis.

Malnutrition caused by dialysis may increase the mortality rate of patients on peritoneal dialysis (Chung et al., 2003). Probiotic capsules containing *B. longum*, *Lactobacillus bulgaricus*, and *S. thermophilus* can promote gastrointestinal absorption and digestion, thus improving nutritional indicators such as serum albumin levels, upper arm circumference, and triceps skinfold thickness. Additionally, they can reduce inflammatory indicators such as serum hypersensitive C-reactive protein and IL-6 (Pan et al., 2021). The increase in inflammatory factor IL-6 not only leads to malnutrition in patients on peritoneal dialysis but also changes the peritoneal small solute transport rate, thereby increasing the mortality rate of patients on peritoneal dialysis (Lambie et al., 2013). *Lactobacillus casei Zhang* can significantly increase beneficial bacteria that produce SCFAs, such as *Dubosiella*, Lachnospiraceae, *Parvibacter*, and *Butyricicoccus*, in the intestines of mice. This increase promotes the production of butyrate and subsequently activates and suppresses the proliferator-activated receptor-γ and nuclear factor-κB pathways, respectively. Activation of these pathways helps to reduce the infiltration of macrophages, polarization toward an inflammatory M1 phenotype, and release of inflammatory cytokines in the peritoneal dialysis effluent, thereby alleviating inflammation in the peritoneum and preventing the progression of peritoneal fibrosis (Wu Z. et al., 2023) (Figure 3).

The types and quantities of substrates that reach the human colon are crucial to the improvement of the gut microbiome composition and metabolism. Therefore, improving uremic toxins in patients on peritoneal dialysis with the use of prebiotics requires high patient compliance to ensure adequate and timely intake and appropriate use. A randomized controlled trial proved that patients on peritoneal dialysis could improve their serum IS levels only with the intake of an adequate amount (21 g/d) of unripe banana flour (de Andrade et al., 2021). Insulin-type fructan can reduce *Bacteroides thetaiotaomicron*, which produces IS, thus inhibiting tryptophanase activity and reducing the production of IS in the gut of patients on peritoneal dialysis. However, the production of uremic-related symptoms in patients on peritoneal dialysis is more commonly related to the levels of blood uremic toxins; therefore, further research is needed to determine the beneficial effects of insulin-type fructan on the quality of life of patients (Li et al., 2020). Although insulin-type fructan reduces the production of IS in the gut, it does not reduce the levels of circulating TMAO, possibly because TMAO-producing bacteria are a type of fermentation substrate (Xiong et al., 2023).

Uric acid, a metabolic waste product of arginine metabolism, is associated with an increased risk of death among patients with ESRD (especially male patients with ESRD) and higher blood uric acid levels, which may be related to higher cardiovascular mortality rates (Xia et al., 2014; Hassan et al., 2022). Insulin-type prebiotics can enrich purine-degrading bacteria, such as *Clostridiales* and *Clostridium* species, which can increase fecal uric acid degradation and reduce blood uric acid concentrations in patients on peritoneal dialysis (He S. et al., 2022).

Peritonitis associated with peritoneal dialysis can alter the structure and function of the peritoneum, leading to fibrosis and, ultimately, the loss of its ultrafiltration capabilities (Perl et al., 2020; Masola et al., 2022). Additionally, for severe cases of toxicity related to p-cresol or para-cresol sulfates, the use of synbiotics can boost the growth of *Bifidobacterium bifidum* strains, enhance the presence of *Lactobacillus* in the gut microbiota, and decrease the levels of p-cresol within the intestine (Stuivenberg et al., 2022).

### 6.2 Inhibitory effects of natural medicines on peritoneal fibrosis

Patients on peritoneal dialysis who use natural medicines can relatively specifically reduce certain gut bacteria and uremic toxins. Most bacteria that produce PCS in the gut are Gram-positive. Berberine can produce an antibiotic-like effect that specifically reduces the abundance of certain Gram-positive gut bacteria such as *Clostridium* species, thereby inhibiting the tyrosine–p-cresol pathway and reducing plasma p-cresol concentrations, thus significantly improving the kidney function. However, unlike other antibiotic groups, berberine can increase the abundance of butyrate-producing bacteria and the fecal butyrate content, thus reducing the deterioration of the kidney function (Pan et al., 2023).

For patients on peritoneal dialysis, peritoneal fibrosis leads to the transition from peritoneal dialysis to hemodialysis. Certain natural medicines can delay this, however. FuShen granules can significantly delay the progression of peritoneal fibrosis by inhibiting the expressions of IL-6, transforming growth factor-β1, connective tissue growth factor, and vascular endothelial growth factor, increasing the abundance of *Bacteroides*, promoting the production of SCFAs, maintaining homeostasis of the colonic mucosal barrier, and promoting the absorption of nutrients in the gut (Lin W. et al., 2021). Nevertheless, more studies are needed to determine the safety and efficacy of FuShen granules.

## 7 Outlook

The key to dialysis treatment is the removal of uremic toxins and excess water in the body. The gut microbiome is the main producer of uremic toxins. A full understanding of the gut microbiome is helpful to obtaining a thorough understanding of the pathological process of uremic toxin accumulation and reducing the impact of uremic toxins on patients. CVDs and peritonitis associated with hemodialysis and peritoneal dialysis are extremely common; however, the monitoring methods for these complications are relatively outdated, and diagnosis is only possible after the onset of disease (Nataatmadja et al., 2016). The composition of the gut microbiome changes when patients on hemodialysis and peritoneal dialysis develop CVDs or peritonitis. Because of the advancement of detection methods, changes in the gut microbiome may play a role in predicting the complications of hemodialysis and peritoneal dialysis.

Patients on dialysis frequently accumulate uremic toxins, thus reducing their quality of life and increasing the probability of complications. Studies have indicated that probiotics, prebiotics, and natural medicines can reduce the accumulation of uremic toxins, thus reducing the incidence of complications and alleviating the symptom burden of patients on dialysis (Vanholder et al., 2016; Huang et al., 2021; Nguyen et al., 2021). Although relatively small-scale clinical research of microbiomes has been performed, further research may establish the use of probiotics, prebiotics, natural medicines, and metabolic products to improve the gut microbiome and effectively mitigate the occurrence of complications.
